# Acquired modification of sphingosine-1-phosphate lyase activity is not related to adrenal insufficiency

**DOI:** 10.1186/s12883-018-1049-9

**Published:** 2018-04-23

**Authors:** Gulin Sunter, Ece Oge Enver, Azad Akbarzade, Serap Turan, Pinar Vatansever, Dilek Ince Gunal, Goncagul Haklar, Abdullah Bereket, Kadriye Agan, Tulay Guran

**Affiliations:** 1 Department of Neurology, Marmara University, Istanbul, Turkey; 2 Department of Paediatric Endocrinology and Diabetes, Marmara University, Fevzi Cakmak Mh. Mimar Sinan Cd.No 41., Ustkaynarca/Pendik, 34899 Istanbul, Turkey; 3 Department of Biochemistry, Marmara University, Istanbul, Turkey

**Keywords:** Sphingosine-1-phosphate lyase, Fingolimod, Adrenal, Multiple sclerosis

## Abstract

**Background:**

Congenital sphingosine-1-phosphate (S1P) lyase deficiency due to biallelic mutations in *SGPL1* gene has recently been described in association with primary adrenal insufficiency and steroid-resistant nephrotic syndrome. S1P lyase, on the other hand, is therapeutically inhibited by fingolimod which is an oral drug for relapsing multiple sclerosis (MS). Effects of this treatment on adrenal function has not yet been evaluated. We aimed to test adrenal function of MS patients receiving long-term fingolimod treatment.

**Methods:**

Nineteen patients (14 women) with MS receiving oral fingolimod (Gilenya®, Novartis) therapy were included. Median age was 34.2 years (range; 21.3–44.6 years). Median duration of fingolimod treatment was 32 months (range; 6–52 months) at a dose of 0.5 mg/day. Basal and ACTH-stimulated adrenal steroid measurements were evaluated simultaneously employing LC-MS/MS based steroid panel. Basal steroid concentrations were also compared to that of sex- and age-matched healthy subjects. Cortisol and 11-deoxycortisol, 11-deoxycorticosterone and dehydroepiandrosterone were used to assess glucocorticoid, mineralocorticoid and sex steroid producing pathways, respectively.

**Results:**

Basal ACTH concentrations of the patients were 20.8 pg/mL (6.8–37.8 pg/mL) (normal range; 5–65 pg/mL). There was no significant difference in the basal concentrations of cortisol, 11-deoxycortisol, 11-deoxycorticosterone and dehydroepiandrosterone between patients and controls (*p* = 0.11, 0.058, 0.74, 0.15; respectively). All patients showed adequate cortisol response to 250 mcg IV ACTH stimulation (243 ng/mL, range; 197–362 ng/mL). There was no significant correlation between duration of fingolimod treatment and basal or ACTH-stimulated cortisol or change in cortisol concentrations during ACTH stimulation test (*p* = 0.57, 0.66 and 0.21, respectively).

**Conclusion:**

Modification and inhibition of S1P lyase activity by the long-term therapeutic use of fingolimod is not associated with adrenal insufficiency in adult patients with MS. This suggests that S1P lyase has potentially a critical role on adrenal development rather than the function of a fully mature adrenal gland.

## Background

The essential role of sphingolipid metabolism has recently been emerged in adrenal disease. Three research groups almost simultaneously reported that recessive mutations in *SGPL1*, which encodes sphingosine-1-phosphate (S1P) lyase, cause a syndromic form of steroid-resistant nephrotic syndrome with adrenal insufficiency [1–3]. S1P lyase is the enzyme responsible for irreversible S1P degradation which is the final step of sphingolipid breakdown.

Sphingolipids are integral components of cell membranes in the regulation of fluidity and lipid bilayer composition, organizing the assembly of signaling molecules, and protein trafficking. Particularly, some sphingolipids like sphingosine (Sph), sphingosine-1-phosphate (S1P), and ceramide (Cer) also act as bioactive signaling molecules and are responsible for the regulation of cell growth, differentiation, senescence, or apoptosis [4].

Given a considerable role in health and disease, S1P signalling system has been extensively studied for the treatment of various inflammatory and autoimmune diseases over the last two decades. Particularly S1P lyase is still a promising target for the treatment of inflammatory and autoimmune diseases.

FTY720 (fingolimod) was designed by modification of myriocin, a naturally occurring sphingoid base analog that causes immunosuppression by interrupting sphingolipid metabolism. It was approved by the U.S. Food and Drug Administration (FDA) in 2010 for adults with relapsing forms of multiple sclerosis (MS) to reduce the frequency of clinical relapses and to delay physical disability [5].

Fingolimod has been shown to inhibit S1P lyase activity in vitro and in vivo [6, 7]. However, long-term effects of this treatment on adrenal function has not been studied so far.

Here, we have tested the adrenal function of the patients under long-term fingolimod (Gilenya®, Novartis) treatment due to relapsing-remitting MS. We aimed to explore whether therapeutic S1P receptor modulation and/or S1P lyase inhibition lead to any impairment in adrenal function on long-term.

## Methods

Study was performed with the approval of the Ethics Committee of Marmara University, Faculty of Medicine, Istanbul, Turkey (09.2016.641). Patients provided written informed consent, and all studies were conducted in accordance with the principles of the Declaration of Helsinki.

We studied 19 patients (14 women, 5 men) with MS receiving oral fingolimod (Gilenya®, Novartis) therapy. Median age was 34.2 years (range; 21.3–44.6 years). Median duration of fingolimod treatment was 32 months (range; 6–52 months) at a dose of 0.5 mg/day. None of the patients was on daily steroid treatment, they had been treated with systemic steroids only during exacerbations. However, none of the patients required such treatment at least a year preceding the study.

Fifteen healthy controls (8 women, 7 men) aged from 27 to 46 years (median; 31.5 yrs) were selected randomly among the hospital staff who gave consent to contribute into the study. Baseline samples of healthy controls were collected at similar conditions as the patients’ to compare normal basaline adrenal steroid secretion. Healthy controls had unremarkable past medical history and had normal blood pressure and physical examination.

We excluded patients and controls who used any anabolic medication, corticosteroids, sex steroids, or gonadotropins.

### Protocol

Both patients and healthy controls provided basal blood samples between 8:00 and 10:00 AM. Then adrenocorticotrophin (ACTH) stimulation test (high dose synacthen test, HDST) was performed to the patients between 08:00 and 10:00 h. Blood samples were obtained by venipuncture before and 60 min after the application of IV 250 μg of synacthen (Novartis Pharma) [8].

All the plasma steroids were assayed simultaneously in patients and control subjects by means of liquid chromatography-mass spectrometry (LC-MS/MS).

### LC-MS/MS serum steroid assays

Plasma concentrations of 17 steroid hormones (17OH-Progesterone, 21-deoxycortisol, androstenedione, dehydroepiandrosterone-sulphate (DHEA-S), dehydroepiandrosterone (DHEA), testosterone, cortisol, cortisone, corticosterone, aldosterone, 11-deoxycortisol, dihydrotestosterone, androsterone, pregnenolone, 17OH-pregnenolone, progesterone, 11-deoxycorticosterone) comprising mineralocorticoids, glucocorticoids and androgens, were determined using LC-MS/MS 8050 system (Schimadzu, Japan). Whole blood samples were collected in K2 EDTA containing tubes (Becton Dickenson, USA). Plasma samples were aliquoted and kept frozen in − 20 °C degrees until the day of analysis. Internal standard mixtures which included 3 different internal standards namely the deuterated forms of aldosterone (aldo d7), cortisol (corti d4), testosterone (testo d3) for the determination of mineralocorticoids, glucocorticoids and androgens were added to each plasma sample, calibrator and control material to monitor recovery. All samples were extracted with S.r.l. Steroid Hormones kit (Eureka Lab Division, Italy). To separate substances an HPLC method was used with a RRHD Eclipse Plus C18 column (50 × 2.1 mm, 1.8 um) at a total flow rate of 0,4 mL/min at 60 °C. Total running time is 15 min and the injection volume was 20 uL. Electrospray ionization (positive mode) was used and for each hormone two multiple reaction monitoring (MRM) were recorded.

### Statistical analysis

Statistical evaluation was performed using GraphPad Prism® V5.0 software (GraphPad Software Inc., San Diego, California, USA). The results for each steroid are reported as median (range) in the text. Mann-Whitney U and Spearman correlation tests were used for comparison and correlation analyses, respectively. Values were considered statistically significant when *P* value is less than 0.05.

## Results

The ages of patient and control groups were similar (*p* = 0.63). Ages of men and women subgroups in patient and control groups were also similar (*p* = 0.76 and 0.24, respectively).

None of the patients showed proteinuria or lymphopenia (data not shown).

**Fig. 1:**
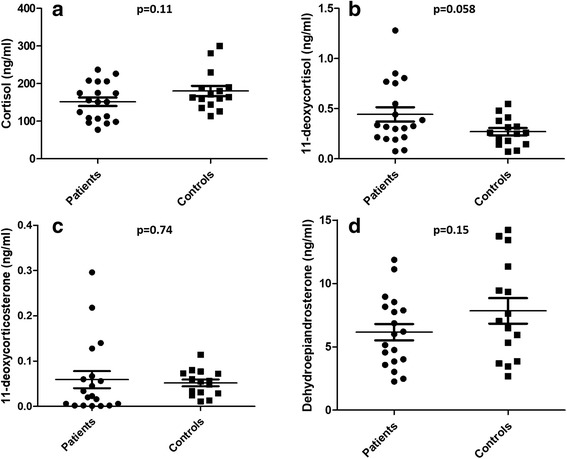
LC–MS/MS based measurement of basal plasma steroids. Cortisol (**a**), 11-deoxycortisol (**b**), 11-deoxycorticosterone (**c**) and dehydroepiandrosterone (DHEA) (**d**) concentrations in patients with multiple sclerosis receiving fingolimod treatment and healthy controls are compared (*p* = 0.11, 0.058, 0.74, 0.15; respectively). Scatter plots represent the mean and standard errors of mean (S.E.M) of the measurements. Each symbol represents an individual case-specific measurement. Conversion to SI units: to convert nanograms per milliliter to nanomoles per liter, multiply by 2.76 for cortisol, by 2.89 for 11-deoxycortisol, by 3.03 for 11-deoxycorticosterone and by 3.467 for DHEA

**Fig. 2:**
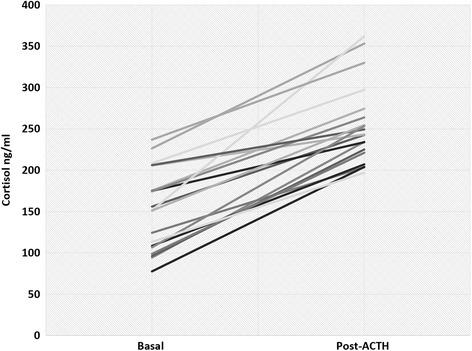
LC–MS/MS based determination of basal- and ACTH-stimulated plasma concentrations of cortisol in patients with multiple sclerosis receiving fingolimod treatment. Conversion to SI units: to convert nanograms per milliliter to nanomoles per liter, multiply by 2.76 for cortisol

Basal ACTH concentrations of the patients were 20.8 pg/mL (6.8–37.8 pg/mL) (normal range; 5–65 pg/mL). There was no significant difference in the basal concentrations of cortisol, aldosterone, dehydroepiandrosterone (DHEA), dehydroepiandrosterone sulphate (DHEAS), 11-deoxycortisol, 11-deoxycorticosterone and other key steroids in adrenal steroid pathways between patients and controls (Table 1). Despite MS patients show some variability in the measurements of 11-deoxycortisol and 11-deoxycorticosterone, median and mean values were still within the normal range of respective parameters (Fig. 1). All patients showed adequate cortisol response to ACTH stimulation (243 ng/mL, range; 197–362 ng/mL) (Fig. 2) [8].

**Table 1:** Comparison of basal steroid measurements of the MS patients with fingolimod treatment and healthy subjects

Parameter	Patients (*n* = 19) median (range)	Healthy controls (*n* = 15) median (range)	*P* value
Age (yrs)	34.2 (21.3–44.6)	31.5 (27–46)	0.63
Basal plasma cortisol (ng/ml) (N: 50–250 ng/ml)	151 (77–237)	164 (113–300)	0.11
Basal plasma aldosterone (N: < 0.24 ng/ml)	0.064 (0.023–0.20)	0.1 (0.04–0.21)	0.38
Basal plasma DHEA (ng/ml) (M: 0.61–16.3 ng/ml F: 1.02–11.85 ng/ml)	6 (2.2–11.8)	7 (2.6–14.2)	0.15
Basal plasma DHEA-S (ng/ml) (M: 480–3340 ng/ml F: 440–3220 ng/ml)	1083 (779–2825)	1427 (572–3110)	0.22
Basal plasma 11-deoxycortisol (ng/ml) (M: 0.14–1.2 ng/ml F: 0.17–1.2 ng/ml)	0.32 (0.07–1.28)	0.25 (0.07–0.54)	0.058
Basal plasma 11-deoxycorticosterone (ng/ml) (M: ≤0.15 ng/ml F: ≤0.18 ng/ml)	0.02 (0.001–0.29)	0.05 (0.01–0.11)	0.74
Basal plasma 17OH- pregnenolone (ng/ml) (M: < 1.28 ng/ml F: < 9.09 ng/ml)	1.3 (0.14–7.6)	0.52 (0.16–3.84)	0.28
Basal plasma 17OH- progesterone (M: < 2.20 ng/ml F: < 2.85 ng/ml)	0.98 (0.20–1.94)	1.25 (0.18–2.59)	0.09
Basal plasma progesterone (M: < 0.20 ng/ml F: 2.7–31 ng/ml)	0.07 (0.006–13.85)	0.08 (0.01–21.8)	0.27
Basal plasma pregnenolone (N: ≤ 3.25 ng/ml)	0.52 (0.16–2.25)	0.77 (0.42–1.12)	0.79
Basal plasma 21- deoxycortisol (N: < 0.5 ng/ml)	0.06 (0.02–0.12)	0.08 (0.03–0.31)	0.89
Basal plasma androstenedione (N: 0.20–2.50 ng/ml)	0.79 (0.32–1.50)	1.06 (0.23–2.2)	0.26

There was no significant correlation between duration of fingolimod treatment and basal or ACTH-stimulated cortisol or change in cortisol concentrations during ACTH stimulation test (*p* = 0.57, 0.66 and 0.21, respectively).

## Discussion

Congenital sphingosine-1-phosphate (S1P) lyase deficiency due to biallelic mutations in *SGPL1*, has recently been established as a cause of primary adrenal insufficiency and steroid-resistant nephrotic syndrome [1–3]. In this study, however, we have shown that therapeutic inhibition and modification of S1P lyase activity does not impair adrenal steroid secretion in patients with multiple sclerosis.

Cortisol is a key regulator of the immune system, energy metabolism, and stress and it is well-known that psychosocial stress has frequently been associated with disease activity and acute exacerbations in MS. The hypothalamus-pituitary-adrenal (HPA) axis is generally activated in MS patients and is associated with disease progression. High cortisol levels were associated with slower disease progression, whereas patients with low cortisol levels had greater numbers of active lesions [9]. Furthermore, patients with shorter disease duration display higher cortisol stress response while MS patients with longer disease duration showed a significantly diminished HPA response. However, a recent study showed that relapsing-remitting MS patients did not differ in stress-related cortisol/catecholamine levels and glucocorticoid sensitivity than control subjects [10]. Despite these findings, very little is known about general adrenal activity and how this function is affected by current medical therapies in MS.

S1P lyase mediates the final step of the sphingolipid metabolism which is the irreversible breakdown of S1P to ethanolamine phosphate and hexadecenal [4]. Inhibition of S1P lyase activity will lead to accumulation of bioactive signaling molecules upstream of the pathway including S1P and ceramides (Cer). Cellular stress and changes in the cellular redox status can further aggravate this toxic accumulation [11, 12]. We have recently demonstrated that accumulation of S1P, Cer and potentially other upstream components of sphingolipid pathway due to congenital S1P lyase deficiency lead to a multisystemic disorder including primary adrenal insufficiency, nephrotic syndrome and ichthyosis [1].

Although the novel role of sphingolipid metabolism on adrenal function has recently been described, the anabolic, catabolic, and signaling pathways of sphingolipids have already emerged as promising targets for the treatment of diverse autoimmune disorders over the last two decades. Especially the introduction of fingolimod into market as first oral drug for the treatment of MS has boosted this effect [4, 5].

FTY720 (Fingolimod, 2-amino-2[2-(4-octylphenyl)ethyl]-1,3-propanediol) is a synthetic analogue of sphingosine. Fingolimod is phosphorylated by sphingosine kinase 2 to generate phospho-fingolimod. Phospho-fingolimod causes the internalization of S1P receptors, which sequesters lymphocytes in lymph nodes, preventing them from moving to the central nervous system and cause a relapse in MS.

Inhibition of S1P lyase is another mechanism of action of fingolimod besides modulation of S1P receptor activity. Fingolimod has been shown to inhibit S1P lyase activity in vitro and in vivo. Bandhuvula P et al. showed that the treatment with fingolimod inhibited tissue S1P lyase activity in mice and in HEK293 cells [6]. Park SM et al. further demonstrated that CD68(+) antigen presenting cells generated from human monocytes were able to internalize and irreversibly degrade S1P, and this activity was inhibited by the S1P analogue fingolimod [7]. This body of evidence warranted to explore the potential long-term effects of fingolimod treatment on adrenal function. A recent study has shown a good safety and efficacy profile of fingolimod over a 36-month treatment [13]. We have particularly confirmed that there is no impairment in adrenal steroid secretion up to 52-month of fingolimod treatment. Our patients did not also show proteinuria or lymphopenia all of which can be seen in patients with congenital S1P lyase deficiency (data not shown).

Adrenal glands of *Sgpl1−/−* mice show compromised zonation between zona glomerulosa (ZG) and zona fasciculate (ZF), and between ZF and X-zone. They also have lower expression of cytochrome P450 side-chain cleavage (CYP11A1), which facilitates the first and rate-limiting step of steroidogenesis [1]. Congenital S1P lyase deficiency causes adrenal insufficiency in early life in humans. Patients present with glucocorticoid, mineralocorticoid and sex steroid deficiency. These data suggest that *SGPL1* may have a developmental role in human adrenal. In our study, however, we tested adult human adrenal function under acquired modification and inhibition of S1P lyase activity. The use of LC-MS/MS based steroid panel enabled us to evaluate the glucocorticoid, mineralocorticoid and sex steroid producing pathways simultaneously. Concentrations of cortisol and 11-deoxycortisol, 11-deoxycorticosterone and dehydroepiandrosterone representing the secretion of zona fasciculata, zona glomerulosa and reticularis, respectively, were similar with healthy controls. Furthermore, all of the patients on fingolimod treatment showed sufficient cortisol response to ACTH stimulation regardless of treatment duration. Our data may suggest that the effect of genetic deficiency of SGPL1 must be related to developmental effects on the adrenal gland. While this may be true, especially in view of the disordered zonation observed in the SGPL1-deficient mice, it seems possible that additional effects of fingolimod, e.g., its agonist activity towards S1P receptors once it is phosphorylated, could also compensate for any ability of fingolimod to suppress adrenal steroid production through its inhibition of SGPL1.

## Conclusion

In conclusion, modification and inhibition of S1P lyase activity by the long-term therapeutic use of fingolimod is safe for the adrenal function in adult patients with MS. These findings support that S1P lyase may have a critical role on adrenal development rather than the function of a fully mature adrenal gland.

## Abbreviations

ACTH: Adrenocorticotrophin
Cer: Ceramide
DHEA: Dehydroepiandrosterone
DHEA-S: Dehydroepiandrosterone-sulphate
LC-MS/MS: Liquid chromatography-mass spectrometry
S1P: Sphingosine-1-phosphate
Sph: Sphingosine

## References

[1] Prasad R, Hadjidemetriou I, Maharaj A, Meimaridou E, Buonocore F, Saleem M, Hurcombe J, Bierzynska A, Barbagelata E, Bergadá I, Cassinelli H, Das U, Krone R, Hacihamdioglu B, Sari E, Yesilkaya E, Storr HL, Clemente M, Fernandez-Cancio M, Camats N, Ram N, Achermann JC, Van Veldhoven PP, Guasti L, Braslavsky D, Guran T, Metherell LA (2017). Sphingosine-1-phosphate lyase mutations cause primary adrenal insufficiency and steroid-resistant nephrotic syndrome. J Clin Invest.

[2] Lovric S, Goncalves S, Gee HY, Oskouian B, Srinivas H, Choi WI, Shril S, Ashraf S, Tan W, Rao J, Airik M, Schapiro D, Braun DA, Sadowski CE, Widmeier E, Jobst-Schwan T, Schmidt JM, Girik V, Capitani G, Suh JH, Lachaussée N, Arrondel C, Patat J, Gribouval O, Furlano M, Boyer O, Schmitt A, Vuiblet V, Hashmi S, Wilcken R, Bernier FP, Innes AM, Parboosingh JS, Lamont RE, Midgley JP, Wright N, Majewski J, Zenker M, Schaefer F, Kuss N, Greil J, Giese T, Schwarz K, Catheline V, Schanze D, Franke I, Sznajer Y, Truant AS, Adams B, Désir J, Biemann R, Pei Y, Ars E, Lloberas N, Madrid A, Dharnidharka VR, Connolly AM, Willing MC, Cooper MA, Lifton RP, Simons M, Riezman H, Antignac C, Saba JD, Hildebrandt F (2017). Mutations in sphingosine-1-phosphate lyase cause nephrosis with ichthyosis and adrenal insufficiency. J Clin Invest.

[3] Janecke AR, Xu R, Steichen-Gersdorf E, Waldegger S, Entenmann A, Giner T, Krainer I, Huber LA, Hess MW, Frishberg Y, Barash H, Tzur S, Schreyer-Shafir N, Sukenik-Halevy R, Zehavi T, Raas-Rothschild A, Mao C, Müller T (2017). Deficiency of the sphingosine-1-phosphate lyase SGPL1 is associated with congenital nephrotic syndrome and congenital adrenal calcifications. Hum Mutat.

[4] Vogt D, Stark H (2017). Therapeutic strategies and pharmacological tools influencing S1P signaling and metabolism. Med Res Rev.

[5] Brinkmann V, Billich A, Baumruker T, Heining P, Schmouder R, Francis G, Aradhye S, Burtin P (2010). Fingolimod (FTY720): discovery and development of an oral drug to treat multiple sclerosis. Nat Rev Drug Discov.

[6] Bandhuvula P, Tam YY, Oskouian B, Saba JD (2005). The immune modulator FTY720 inhibits sphingosine-1-phosphate lyase activity. J Biol Chem.

[7] Park SM, Angel CE, McIntosh JD, Brooks AE, Middleditch M, Chen CJ, Ruggiero K, Cebon J, Rod Dunbar P (2014). Sphingosine-1-phosphate lyase is expressed by CD68+ cells on the parenchymal side of marginal reticular cells in human lymph nodes. Eur J Immunol.

[8] Stewart PM, Krone N, Melmed S, Polonsky KS, Larsen PR, Kronenberg HM (2011). The adrenal cortex. Williams textbook of endocrinology.

[9] Melief J, de Wit SJ, van Eden CG, Teunissen C, Hamann J, Uitdehaag BM, Swaab D, Huitinga I (2013). HPA axis activity in multiple sclerosis correlates with disease severity, lesion type and gene expression in normal-appearing white matter. Acta Neuropathol.

[10] Kern S, Rohleder N, Eisenhofer G, Lange J, Ziemssen T (2014). Time matters - acute stress response and glucocorticoid sensitivity in early multiple sclerosis. Brain Behav Immun.

[11] Goldkorn T, Balaban N, Shannon M, Chea V, Matsukuma K, Gilchrist D (1998). H2O2 acts on cellular membranes to generate ceramide signaling and initiate apoptosis in tracheobronchial epithelial cells. J Cell Sci.

[12] Liu B, Andrieu-Abadie N, Levade T, Zhang P, Obeid LM, Hannun YA (1998). Glutathione regulation of neutral sphingomyelinase in tumor necrosis factor-alpha-induced cell death. J Biol Chem.

[13] Saida T, Itoyama Y, Kikuchi S, Hao Q, Kurosawa T, Ueda K, Auberson LZ, Tsumiyama I, Nagato K, Kira JI (2017). Long-term efficacy and safety of fingolimod in Japanese patients with relapsing multiple sclerosis: 3-year results of the phase 2 extension study. BMC Neurol.

